# Inner speech-related brain areas mapped using a novel rhyming task

**DOI:** 10.3389/fpsyg.2026.1856832

**Published:** 2026-09-09

**Authors:** Lucila Barbosa, Pablo del Olmo-Encabo, Ana Aquino-Servín, Alejandro Sotero, Noemí Hostalet, Alfredo Felices, Irene París-Gómez, Mariona Latorre-Guardia, Betül Yildirim, Emilio J. Inarejos-Clemente, Andriana Karuk, Olivia Gawel, Pilar Salgado-Pineda, Peter J. McKenna, Edith Pomarol-Clotet, Paola Fuentes-Claramonte

**Affiliations:** 1 FIDMAG Germanes Hospitalàries Research Foundation, Barcelona, Spain; 2 Doctoral Program in Biomedicine, Universitat de Barcelona (UB), Barcelona, Spain; 3 Doctoral Program in Cognitive Science and Language, Universitat de Barcelona (UB), Barcelona, Spain; 4 Network Centre for Biomedical Research on Mental Health (CIBERSAM), Instituto de Salud Carlos III, Madrid, Spain; 5 Departament de Biologia Evolutiva, Ecologia i Ciències Ambientals (BEECA), Facultat de Biologia, Universitat de Barcelona (UB), Barcelona, Spain; 6 Fundació Hospitalàries Sant Boi, Sant Boi de Llobregat, Spain; 7 Doctoral Program in Psychiatry, Universitat Autònoma de Barcelona, Bellaterra, Spain; 8 Servicio de Diagnóstico por la Imagen, Hospital Sant Joan de Déu (HSJD), Barcelona, Spain; 9 Department of Cognitive and Information Sciences, University of California, Merced, Merced, CA, United States; 10 Health Sciences Research Institute, University of California, Merced, Merced, CA, United States

**Keywords:** Broca's area, fMRI, inferior frontal cortex, phonological encoding, rhyming

## Abstract

**Introduction:**

The brain functional correlates of phonological encoding have been examined by means of tasks requiring silent articulation, in many cases utilizing judgments of whether two words rhyme or not. However, most studies have employed written stimuli, which may not be appropriate in languages with transparent orthography (e.g. Spanish), and control tasks have often been less than ideal for functional imaging purposes. We aimed to identify brain activations during phonological encoding using a visual rhyming judgment task that minimized reading-related confounds, and we employed a novel control task that engaged presemantic stages of visual object processing.

**Methods:**

Twenty-eight healthy, right-handed adults, out of an initial sample of 32, performed a task requiring them to judge whether the names of line drawings of animals and objects rhymed. The control task consisted of judging whether two pictures of animals and objects viewed from different angles were the same or different. Brain activations were examined using fMRI and whole-brain, voxel-based analysis.

**Results:**

The rhyming condition (rhyming > control) elicited increased activation in the left inferior frontal cortex, including Broca's area, as well as the precentral, supramarginal, and superior temporal gyri, plus the supplementary motor area, with subcortical activity being seen in the basal ganglia and left cerebellum. The reverse contrast (control > rhyming) activated occipital and ventral temporal cortical regions.

**Discussion:**

These results support the validity of this new picture-based rhyming task to study phonological encoding, and they highlight the role of the left inferior frontal cortex including Broca's area, among other regions, in silent articulation. Regions activated by the control task overlapped those known to be involved in visual object processing.

## 1 Introduction

Phonological encoding, the process of converting abstract word forms into a phonetic plan for how an utterance should be articulated, is important not only from the linguistic perspective, but also for at least two areas of psychological research. One of these is inner speech, the silent articulation of words, phrases, sentences and longer stretches of discourse, an area of significant current investigation, with studies examining to what extent it is universal, the different forms it takes (e.g., monologic and dialogic) and its development during childhood, as well as issues about what exactly it represents (Alderson-Day and Fernyhough, 2015; Dahò and Monzani, 2025). Phonological encoding also plays a role in short-term memory, specifically its verbal component, the phonological or articulatory loop. This maintains limited amounts of verbally coded information in conscious awareness for short periods of time, and combines the action of a passive storage mechanism into which verbal material enters but undergoes rapid decay, with a second process that maintains it in the store by (silent) rehearsal (Baddeley, 1992; Vallar, 2006).

While the brain localization of phonological processing can be and has been examined in patients with brain lesions (Geva et al., 2011; Pillay et al., 2014), key findings in this area have come from functional imaging paradigms designed to induce silent articulation, such as reading, object naming and making judgements about rhyming. This last task takes advantage of the fact that it is known that making rhyming decisions based on visually presented material engages subvocal rehearsal, because it is difficult or impossible to judge whether two words rhyme without doing this (Besner, 1987; Burani et al., 1991; Vallar and Baddeley, 1984; Vallar and Cappa, 1987). The rhyming task (and the closely related task of deciding whether syllables or letters are homophones) also has another advantage, in that it allows subjects' performance to be monitored and measured (Geva et al., 2011).

A pioneering functional imaging study of rhyming was carried out by Paulesu et al. (1993). They used PET combined with whole-brain analysis to examine six healthy subjects while they decided whether single visually presented English letters rhymed with the letter B (which was also displayed on the screen). Compared to a baseline of deciding if Korean letters were similar to a target Korean letter, activation was seen in part of Broca's area (specifically Brodmann Area 44) and its right homolog, and also in the superior temporal gyrus and the insula bilaterally. The authors also found activation in the left supramarginal gyrus, which they suggested might reflect activity in the phonological store component of the articulatory loop. Cerebral areas they presumed were involved in motor aspects of speech, including the supplementary motor area, the premotor cortex, the sensorimotor cortex and the cerebellum, were also activated. A further area of activation was in the left lingual gyrus.

Several further studies examining rhyming-associated brain activations in healthy subjects have been carried out (Lurito et al., 2000; McDermott et al., 2003; Pugh et al., 1996; Owen et al., 2004; Burton et al., 2005; Yen et al., 2019; Liu et al., 2022; Hoeft et al., 2011; Kareken et al., 2000; Poldrack et al., 2001; Seghier et al., 2004). These all used fMRI and had sample sizes ranging from 5 to 45 individuals; some examined activations in predetermined regions of interest (ROIs) (Hoeft et al., 2011; Poldrack et al., 2001; Pugh et al., 1996) whereas others used whole brain analysis (Burton et al., 2005; Kareken et al., 2000; Liu et al., 2022; Lurito et al., 2000; McDermott et al., 2003; Owen et al., 2004; Seghier et al., 2004; Yen et al., 2019). All but two of these studies (Burton et al., 2005; Owen et al., 2004) reported activation in Broca's area.

It is important to note that most of these studies used tasks requiring reading of visually presented pairs of words or non-words, something that could present a problem in languages such as Spanish that have a so-called transparent orthography, i.e., the pronunciation of a word can be deduced from its spelling—in such languages rhyme judgments can be made simply by visually inspecting the orthographic form, without any need for silent pronunciation. (The reverse also applies: if a word is heard, its spelling can be deduced, something that accounts for the fact that there are no spelling contests in Spanish-speaking countries). Another issue related to the use of written words in rhyming tasks is that they will lead to the activation not only of phonology-related brain regions but also those associated with reading.

Use of pictorial as opposed to verbal stimuli avoids both the above confounds. It may also confer advantages from the point of view of the subtractive methodology commonly used in functional imaging research, i.e., using a comparison task that shares cognitive features with the experimental task apart from the one under study. It is widely accepted that processing of visual objects takes place in a series of stages: formation of a two-dimensional initial representation, then a viewer-centered representation, then an object-centered representation that is independent of viewpoint, and finally identification of the object and naming it using semantic memory (Ellis et al., 1988; Farah, 1990; Humphreys and Riddoch, 1987; Marr, 2010). Thus, in principle it should be possible to design control tasks that share many or all of the initial stages of processing with the rhyming task used, and which differ only in not requiring accessing of the semantic and phonological information necessary to enable naming and silent pronunciation.

To date, only one study has employed a control task of this type: MacSweeney et al. (2008) examined hearing and deaf individuals, and also individuals with dyslexia, using a rhyming task (or in the case of the deaf participants, one that required making decisions about whether two British Sign Language signs shared visual articulatory components), presenting two simultaneously presented line drawings of animals and objects. The control task consisted of deciding whether simultaneously presented pairs of pictures were the same or different. While the control task was visual in nature, it is not clear to what extent it would replicate the full range of stages of visual object processing—it could in theory be solved simply by feature comparison without forming a model of what was being seen.

In this study we report fMRI findings using a rhyming task that aimed to incorporate some of the above-identified desirable design features. In the first place, in order to make the task suitable for Spanish and other languages with a transparent orthography (and more generally to be usable internationally), we used a rhyming task based on pictures. Secondly, the control task was designed to engage more of the initial stages of visual object processing than the task used by MacSweeney et al. (2008), but to differ at the final step of naming, and hence ability to make rhyme judgments.

## 2 Methods

### 2.1 Participants

Thirty-two healthy, right-handed adults were recruited for the study, via advertising, social networks and other sources (e.g., word of mouth) in the metropolitan area of Barcelona. Potential participants with a history of significant medical or neurological conditions or head injuries resulting in loss of consciousness were excluded. Other exclusion criteria included current or past substance abuse or dependence, a personal or first-degree family history of major mental illness, or treatment with psychotropic medications. All participants were native or bilingual Spanish speakers (Spanish:Catalan bilingualism is typical in Catalonia).

### 2.2 Ethical approval

All participants gave written informed consent prior to participation. All the study procedures had been previously approved by the FIDMAG Hermanas Hospitalarias Ethical Committee (reference code: PR-2021-15) and adhered to the Declaration of Helsinki. Participants received a gift-card as a compensation for their participation in the study.

### 2.3 Experimental task

Task stimuli for the rhyming and the control task were selected based on a pilot study conducted online with 47 healthy participants (mean age 36.12, SD = 15.83). For the rhyming task, 40 stimulus pairs were generated using grayscale photographs of objects or animals on a white background (21 rhyming and 19 non-rhyming pairs). Participants were asked to (1) judge whether each stimulus pair rhymed or not; (2) write the name of the depicted objects or animals; and (3) rate the affective valence of each stimulus. The selected stimuli were those with the highest agreement in the rhyming and object naming tasks with neutral or positive valence. Twelve rhyming and 12 non-rhyming pairs were selected (for the total of 24 trials in the experimental paradigm). Agreement in the selected pairs ranged from 89.36% to 100%, with an average of 97.96% (SD = 2.77). In the final step, a professional artist converted the grayscale photographs into line drawings for visual uniformity.

The stimuli for the control task consisted of 48 line drawings of animals and objects from different points of view, created by the same professional artist. These drawings were based on those in the Snodgrass and Vanderwart battery (Snodgrass and Vanderwart, 1980), redrawn and supplemented as necessary with original figures.

**Figure 1:**
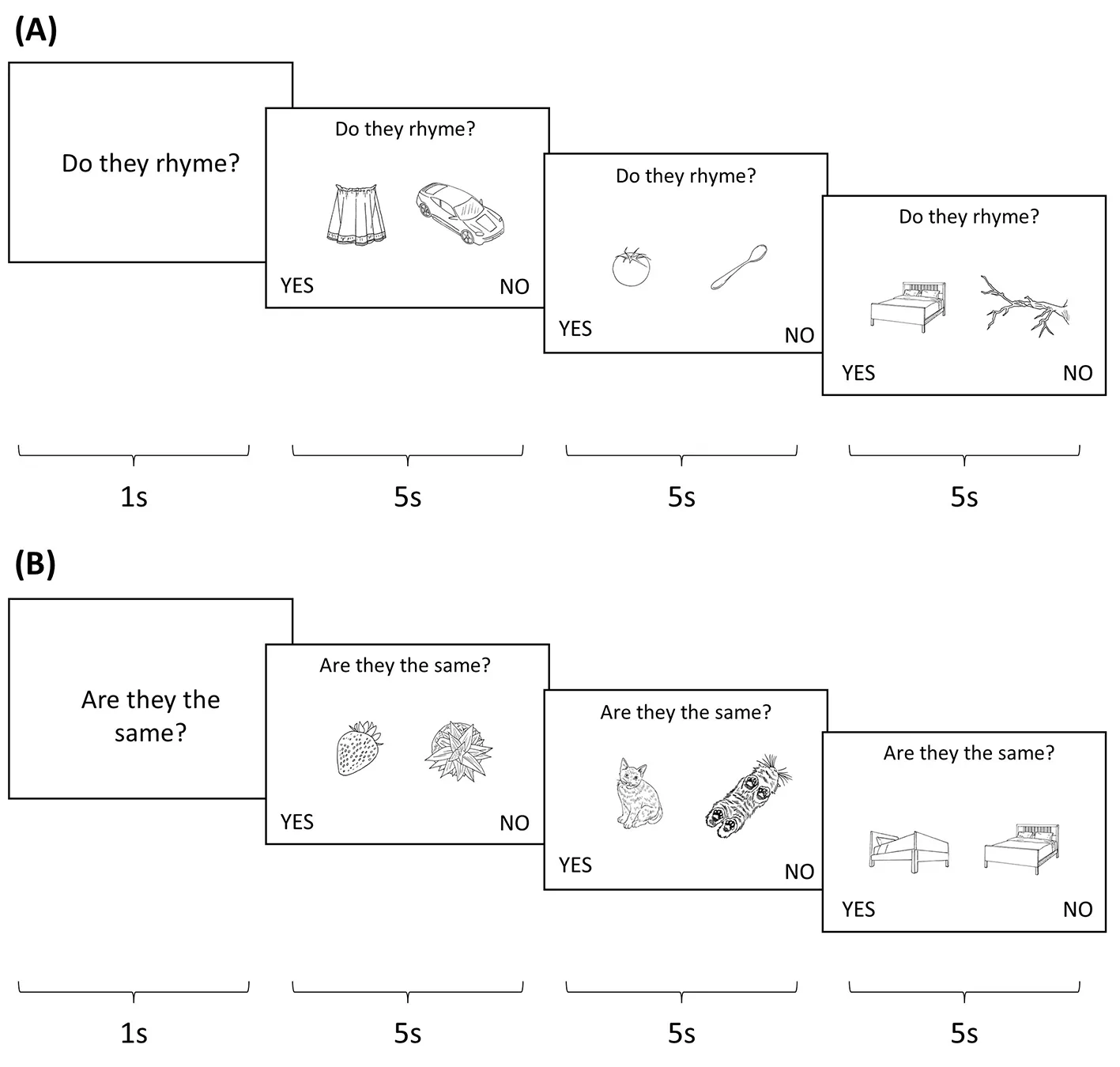
Examples of **(A)** the rhyming task and **(B)** the identity judgment task block. (In the rhyming task only the third pair of objects, *cama* [bed] and *rama* [branch], rhyme in Spanish).

While in the scanner, in the rhyming condition, the participants saw two pictures side by side, and were required to indicate, by a right or left button press, whether the names of the two animals or objects on rhymed or not (for examples see Figure 1A). In the control condition, the participants had to determine whether two side by side pictures, presented from different viewpoints, were of the same animal or object or not, again by a right or left button press (see Figure 1B).

The two conditions were presented in a block-design manner in alternating order up to a total of 8 blocks per condition. Each block consisted of three trials with a duration of 5 s each. An instruction whether to perform the rhyming or same/different judgement task was presented for 1 s immediately before each block began. Between blocks, a fixation cross (a plus symbol) serving as a low-level baseline was presented for 10 s. Total task duration was 8 min and 10 s.

Before scanning, participants underwent a practice session where they learned how to use the response buttons, responded to example stimuli, and familiarized themselves with the task. If bilingual they were instructed to perform the rhyming task in Spanish. Reaction time and accuracy (correct or incorrect response) were recorded for each trial.

### 2.4 Image acquisition

Images were acquired with a 3T Philips Ingenia scanner (Philips Medical Systems, Best, The Netherlands) located in Hospital Sant Joan de Déu (Esplugues de Llobregat, Barcelona, Spain). Functional data were acquired using a T2^*^-weighted echo-planar imaging (EPI) sequence with 242 volumes and the following acquisition parameters: repetition time (TR) = 2000 ms, echo time (TE) = 30 ms, flip angle = 70°, in-plane resolution= 2.5 × 2.5 mm, FOV = 240 × 240 mm, slice thickness = 3.5 mm, inter-slice gap = 0.75 mm. Slices (32 per volume) were acquired with an interleaved order parallel to the AC-PC plane. After the functional sequences, a high-resolution structural T1-weighted sequence was acquired for anatomical reference and inspection, with the following acquisition parameters: matrix size 320 × 320 × 250; voxel resolution 0.75 × 0.75 × 0.8 mm^3^; TR/TE = 8.4/3.8 ms.

### 2.5 Functional MRI preprocessing and analysis

Preprocessing and analysis of the functional data were performed using the FEAT module from the FSL (FMRIB Software Library) package (Smith et al., 2004). The initial 10 s of the sequence (corresponding to five volumes) were excluded to account for signal stabilization. The preprocessing pipeline included motion correction via the MCFLIRT algorithm, followed by co-registration and normalization to a common stereotactic space using the Montreal Neurological Institute (MNI) template. Prior to group-level analyses, normalized functional images underwent spatial smoothing using a Gaussian kernel with a full width at half maximum (FWHM) of 5 mm. To reduce motion artifacts, participants with maximum absolute head movement exceeding 3.0 mm or an average absolute displacement greater than 0.3 mm were excluded from the analysis.

Statistical analysis was performed by means of a General Linear Model (GLM) approach. At the first level, we defined regressors corresponding to the rhyming judgment blocks, the identity judgment blocks, and instruction screens. Six head motion regressors were also added as nuisance covariates. The main contrast of interest for the task was the comparison between the rhyming and control blocks. A supplementary analysis was carried out with trial-wise reaction times added as parametric modulators of the rhyming judgment and identity judgment blocks, to control for task difficulty effects (see Supplementary material). GLMs were fitted to generate individual activation maps for the contrast rhyming > control and second level (group) analyses were performed within the FEAT module by means of mixed-effects GLMs (Beckmann et al., 2003).

Statistical tests were carried out at the cluster level with a corrected *p*-value of 0.05 using Gaussian random field methods. As recommended by Eklund et al. (2016) a conservative threshold of *z* > 3.1, equivalent to an uncorrected *p*-value of < 0.001, was used to define the initial set of clusters.

## 3 Results

### 3.1 Participants

Thirty-two healthy, right-handed adults took part in the study. From this initial sample, 28 (17 male, 11 female) were finally included. Three participants were excluded due to excessive head motion during scanning, and one more was excluded because of missing data (the task was not completed). Fourteen participants were Spanish and the remainder were from other Spanish-speaking countries (Argentina [8], Chile [1], Venezuela [2], others [3]). Their mean age was 38.2 years (SD = 9.29; range = 22–54) and their mean estimated IQ was 102 (SD = 6.67; range = 85–114), as assessed by the Word Accentuation Test (Del Ser et al., 1997; Gomar et al., 2011), a pronunciation test which requires reading aloud Spanish words whose accents have been removed. This test is conceptually similar to the UK National Adult Reading Test (Nelson and Willison, 1991) and the American Wide-Range Achievement Test (Wilkinson and Robertson, 2017). All participants underwent an fMRI session in the same 3T scanner.

### 3.2 Behavioral performance

Reaction time (RT) and accuracy were recorded for each trial. Trials with RT below 100 ms were considered anticipations and were excluded from the analyses. Trials with no responses were also excluded. Overall, 1.3% of the trials were excluded. The maximum number of excluded trials for a single participant was 3.

For the rhyming condition, the mean reaction time was 2505.19 ms (SD = 331.55 ms, range 1882.25–3188.75 ms). The mean proportion of correct responses was 88% (SD = 8%, range 71%−100%). In the control condition, the mean reaction time was 2144.14 ms (SD = 32.81 ms, range 1435.85–2742.35 ms), with a mean accuracy of 86% (SD = 11%, range 54%−100%). Wilcoxon paired signed-rank tests showed significant differences between conditions in RT (*W* = 389, *p* < 0.001, effect size *r* = 0.91) but not in accuracy (*W* = 158, *p* = 0.830, effect size *r* = 0.03).

### 3.3 Task-related activations

**Figure 2:**
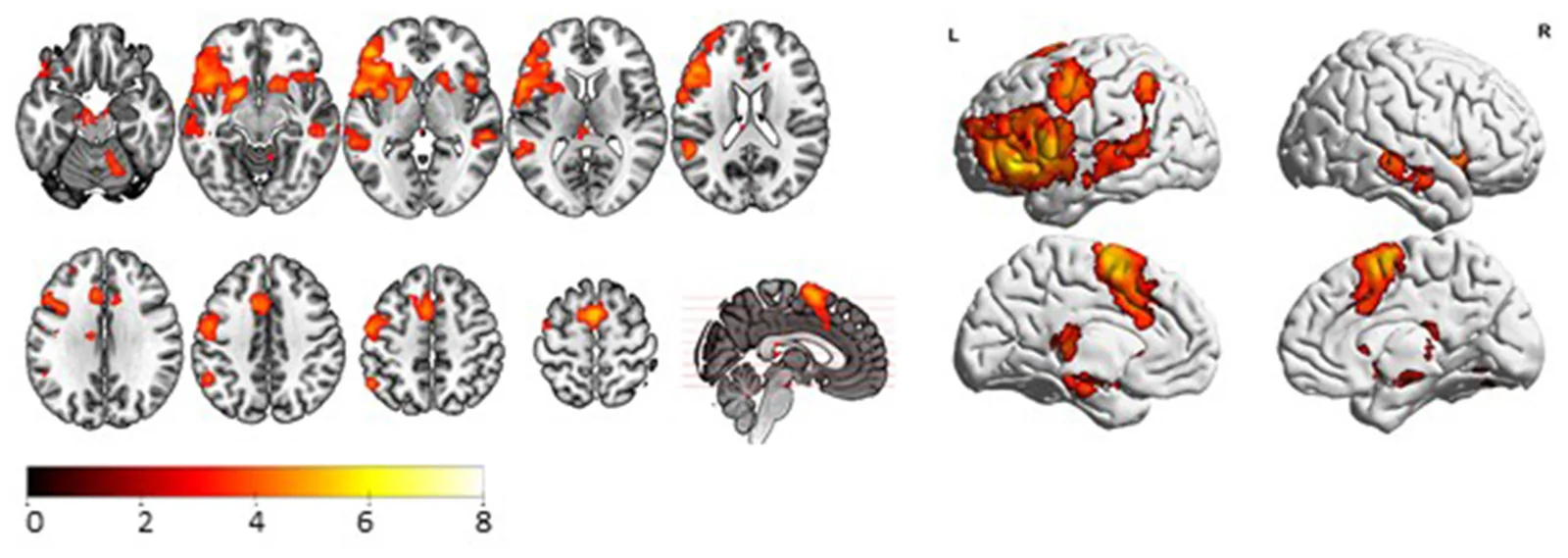
Mean brain activation map for the rhyming > control contrast. Color bar depicts *z*-values. Images are shown in neurological convention (right is right).

Compared to the control condition, the rhyming condition was associated with increased activity in the left inferior frontal cortex, including its three major subdivisions (*pars triangularis, pars opercularis* and *pars orbitalis*), and so encompassing Broca's area, corresponding to the first two of these. This cluster also extended into the anterior insula and Rolandic operculum. Other clusters of activation on the left were seen in the temporal pole and in the posterior superior temporal cortex, supramarginal and inferior parietal cortex, the precentral and postcentral gyri, and the supplementary motor area (SMA), and also the left calcarine cortex. Activation was also observed bilaterally in the middle cingulate gyrus, the anterior insula and the middle temporal gyrus. Subcortically, significant activation was seen in the pallidum, caudate and putamen bilaterally. Additional activation was observed in the right cerebellum (see Table 1 and Figure 2).

**Table 1:** Regions of significant activation in the rhyming > control contrast.

Region	MNI coordinates					
	*x*	*y*	*z*	*Z*	*k*	*p*
Left inferior frontal pars opercularis	−54	32	−6	6.22	9,383	< 0.001
Left inferior frontal pars triangularis	−38	26	2	5.59		
Left putamen	−22	4	−8	5.43		
Left superior temporal pole	−50	12	−2	5.43		
Bilateral supplementary motor area	0	0	66	5.55	2,347	< 0.001
Left middle cingulum	−10	12	44	5.19		
Cerebellum	26	−64	−28	5.17	970	< 0.001
Right putamen	20	16	−6	4.67	798	< 0.001
Right insula	46	16	−2	4.5		
Right caudate	12	12	−10	4.43		
Right middle temporal	56	−34	0	4.54	601	< 0.001
Brainstem	−10	−18	−26	4.24	214	0.004
Brainstem	−2	−28	8	4.11	168	0.014

MNI coordinates indicate peaks of maximum activation.

**Figure 3:**
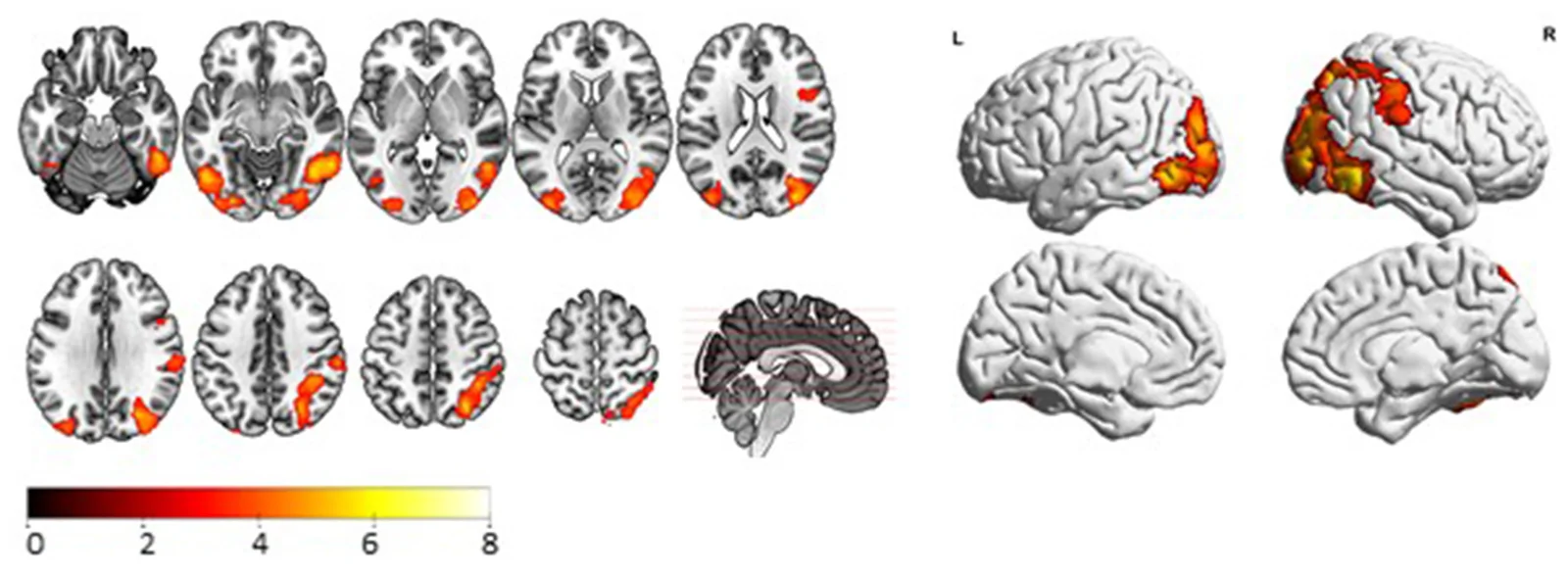
Mean brain activation map for the control > rhyming contrast. Color bar depicts *z*-values. Images are shown in neurological convention (right is right).

We also examined the inverse contrast (control > rhyming). This revealed a pattern of predominantly posterior activation: there were large bilateral clusters involving the inferior and middle occipital cortex, extending into the inferior temporal and fusiform gyri; on the right this cluster extended to reach the right superior occipital cortex and the angular and supramarginal gyri. On the right, there was also a small cluster in the right inferior frontal cortex (*pars opercularis*), (see Table 2 and Figure 3).

**Table 2:** Regions of significant activation in the control > rhyming contrast.

Region	MNI coordinates					
	*x*	*y*	*z*	*Z*	*k*	*p*
Right inferior temporal	50	−54	−14	6.12	7284	< 0.001
Right middle occipital	34	−84	20	5.9		
Right inferior temporal	52	−56	−2	5.64		
Left inferior occipital	−52	−66	−10	5.46	2300	< 0.001
Left middle occipital	−38	−90	8	5.04		
Left fusiform	−42	−72	−14	4.66		
Right precentral	50	4	22	3.99	247	0.001
Right inferior frontal pars opercularis	38	8	22	3.83		

MNI coordinates indicate peaks of maximum activation.

To control for the effect of different RTs in the two task conditions, both analyses were repeated introducing trial-by-trial RTs as a parametric modulator in the first-level analysis (see Supplementary material). After doing this, the activation pattern for the rhyming > control contrast remained similar, although it became more strongly left-lateralized, with no right-sided activation in the anterior insula and basal ganglia now being evident. Left inferior frontal activation was more restricted, and only included the *pars opercularis* and *pars orbitalis*.

In the control > rhyming contrast, the activation map remained essentially the same, although with slightly different cluster sizes (For detailed results, see Supplementary material).

## 4 Discussion

During performance of a task designed to elicit silent articulation, the participants in this study showed prominent activation in a large left-sided area in the left inferior frontal cortex. This included Broca's area, but also extended beyond this to encompass other subdivisions of the inferior lateral frontal cortex and the anterior insula. Task performance was additionally associated with activation in the supplementary motor area extending into the left superior frontal cortex, the insula bilaterally, the middle and superior left temporal cortex, the supramarginal gyrus and inferior parietal cortex, and the right middle temporal cortex. Subcortical activation was seen in the basal ganglia and cerebellum.

Our findings with respect to Broca's area replicate those of most previous studies using rhyming tasks. As far as we can tell there are only two exceptions to this consensus, and both tend to prove the rule. Owen et al. (2004) found no left inferior frontal cortex activation in 8 participants when they indicated whether two pronounceable non-words rhymed or not. However, the control task in this study was speaking aloud nonwords, so that their failure to find left inferior frontal cortex activation during rhyming could be attributable to the fact that the latter task would also be expected to activate Broca's area. Burton et al. (2005) found that deciding whether visually presented words and nonwords rhymed was not associated with left inferior frontal cortex activation in 12 subjects, compared to a control task involving discrimination of two tones as same or different. It should be noted, however, that the analysis in this study was complex and the authors of this study themselves ultimately concluded that greater articulatory recoding demands for processing of nonwords (although not real words) was associated with increased activation in the left inferior frontal cortex.

The study of MacSweeney et al. (2008) referred to in the introduction is also relevant here because it used similar experimental and control paradigms similar to ours. Their seven hearing participants showed a not-dissimilar pattern to the one we found: greater activation in the rhyming vs same:different condition in the left inferior frontal cortex, as well as more dorsal regions of the precentral and lateral prefrontal cortex, and also clusters in the superior portion of the supramarginal gyrus, extending into the superior parietal lobule and medially into the precuneus, the anterior medial portion of the superior frontal gyrus, the anterior cingulate cortex and the right inferior occipital gyrus extending into the fusiform gyrus. Interestingly, the pattern was closely similar in seven deaf individuals.

It is worth noting that functional imaging studies are not the only class of study that has implicated Broca's area/left inferior frontal cortex in rhyming. Geva et al. (2011), examined patients with post-stroke aphasia that affected their production of speech but who had relatively preserved comprehension. The patients performed a rhyming task based on visually presented words as well as another task requiring silent articulation, homophone judgement. Using the technique of lesion mapping, they found that impaired performance on the rhyming task was significantly associated with lesions to an area including Broca's area but extending beyond it through the pre- and postcentral gyrus to the anterior part of the supramarginal gyrus. The findings remained significant for Broca's area and the supramarginal gyrus after controlling for ability to read aloud, and for Broca's area when sentence repetition ability was controlled for. In another non-imaging study, this time carried out on healthy subjects, Gough et al. (2005) found that repetitive magnetic stimulation over a posterior region of the left inferior frontal cortex had a disruptive effect on performance of a rhyming task, significantly increasing response latencies. In contrast, stimulation over a slightly more anterior region instead increased reaction times for performance of a semantic task (deciding if two words were from the same semantic category).

Our findings also implicated the left precentral gyrus and the supplementary motor cortex bilaterally in making rhyming decisions. Both these areas have previously been found to be activated in studies using rhyming tasks (Liu et al., 2022; McDermott et al., 2003; Seghier et al., 2004; Zhuang et al., 2016). The activation seen in these sites probably did not reflect the act of button pressing, since this was also required in the control condition. The premotor cortex has been found to play a role in speech production and perception (Glanz et al., 2018; Hickok et al., 2011). Likewise, the bilateral supplementary motor area has been argued to be implicated in verbal tasks such as speech planning, word retrieval, and speech rehearsal (Hertrich et al., 2016).

We also found a cluster of activation in the left posterior superior temporal cortex, something that has been found in some but not all other rhyming studies (Seghier et al., 2004; Yen et al., 2019; Zhuang et al., 2016). It seems unlikely that activation here reflected the early visual object processing steps culminating in object recognition which take place in this brain region, as these were shared with the control task. However, it could possibly have been related to the naming that is required prior to making decisions about rhyming, and which appears to involve the left posterior temporal regions, albeit among other sites (e.g., see Hamberger and Seidel, 2009; for a summary of research in this area). This cluster of activation in our study also extended into the supramarginal gyrus and inferior parietal cortex, which are known to be involved in articulatory sequencing and integration of sublexical and lexical cues (Deschamps et al., 2014; Oberhuber et al., 2016), and so this also may have been a relevant factor.

More generally, the pattern of rhyming-associated cortical activation we found corresponds in several respects to the dorsal sensorimotor integration pathway described in contemporary dual-stream models of language processing (Friederici, 2011; Chang et al., 2015). These models posit a dorsal pathway, typically left-hemisphere dominant, that conveys phonological information from the posterior superior temporal cortex to premotor and inferior frontal regions, where it is mapped onto articulatory and motor representations. This pathway is complemented by an independent, more bilaterally organized ventral stream that supports speech comprehension and lexical-semantic processing (for a review, see Hickok and Poeppel, 2007). Notably, virtually all regions we found to be activated during the rhyme judgment task, including the left inferior frontal cortex (encompassing Broca's area), the precentral gyrus and supplementary motor area, as well as the posterior superior temporal and supramarginal gyri, correspond to key nodes of this dorsal articulatory-phonological network.

Subcortically, we observed significant activation in the basal ganglia, slightly more extensive on the left, which included the pallidum, caudate, and putamen bilaterally. To the best of our knowledge, these results have not been reported in previous fMRI studies using rhyming tasks, although it is a plausible finding given that the basal ganglia have been previously linked with speech production, especially the left putamen in relation to motor aspects of speech (Zhang et al., 2024). Basal ganglia activation, including the pallidum, caudate, and putamen, has additionally been linked to rhyming and phonological processing through effective connectivity analyses: Booth et al. (2007), using dynamic causal modeling on a visual rhyming task, found that the left putamen showed unidirectional modulatory output to left inferior frontal gyrus and left lateral temporal cortex, which they interpreted as reflecting a role in the cortical initiation of phonological representations, complementing an amplification/refinement role attributed to the cerebellum (Houk et al., 2007). Our findings are also compatible with the broader view that this structure contributes to the phonological assembly of phonemes into speech output (Ullman, 2001).

When we examined the contrast inverse to the one of interest, i.e., same:different judgments > rhyming judgments, we found a circumscribed pattern of mainly posterior activations. This was seen principally in the bilateral occipital cortex (although more extensively on the right) extending anteriorly to include parts of the inferior parietal cortex (angular gyrus), the superior parietal cortex and the inferior temporal cortex, reaching the fusiform gyrus. This pattern is of some theoretical interest as it captures several regions considered to be important for visual object processing, specifically the so-called ventral pathway concerned with identifying the form of a visual stimulus, the “what” as opposed to the “how” or “where” that locates it in space (Grill-Spector and Weiner, 2014; Kravitz et al., 2013; Milner and Goodale, 2008; Ungerleider and Mishkin, 1982). In both humans and non-human primates, this pathway emerges from the primary visual cortex, continues through visual areas (V2, V3, and human V4) and proceeds through a complex and recurrent occipitotemporal network that reaches the anterior temporal lobe (Goodale and Milner, 1992; Kravitz et al., 2013). Why greater activation was found in these regions during object processing than in making rhyming judgments remains conjectural, but a plausible explanation might be that making same:different judgments about objects presented from different perspectives requires focusing more resources in the presemantic stages of visual object processing than does a rhyming task.

Two potential weaknesses to the task we developed need to be acknowledged. Firstly, we cannot rule out that object naming took place during the control condition. Even so, it remains the case that a) naming is not needed to correctly perform the control task, whereas it is for the rhyming task, and b) naming itself does not necessarily imply phonological manipulation of the retrieved name, both of which suggest that at the very least there would have been a greater involvement of articulatory processes in the experimental condition. The second problem concerns the potential influence of geographical differences in object naming in the rhyming condition. For example, the Spanish word “lata” would never be considered to rhyme with the word for money in Spain (“dinero”), but it could do so for a Latin American Spanish speaker (“plata”). Even if this were the case, we do not believe that this would have affected activations to the task, because the purpose of the rhyming task was simply to induce silent articulation, and so it is arguably irrelevant whether the two words silently articulated actually rhymed or not.

In conclusion, we report activation results from a picture-based rather than word-based rhyming task, contrasted with a novel control task designed to allow subtraction of activations related to visual object processing. The notable finding is a large area of activation in the left inferior frontal cortex, including Broca's area but extending beyond it, and so reinforcing the findings of several previous studies using different rhyming and control paradigms. Performance of the rhyming task also resulted in activation in other, predominantly left-sided cortical areas, most of which can be plausibly related to language functions needed for successful making of rhyming decisions.

## Data Availability

The raw data supporting the conclusions of this article will be made available by the authors, without undue reservation.
