# Supplementary material for: Inner speech-related brain areas mapped using a novel rhyming task
Source: Front Psychol. 2026 Sep 9;17:1856832. doi: 10.3389/fpsyg.2026.1856832 (PMC13597393; doi:10.3389/fpsyg.2026.1856832)
Supplement: Supplementary file 1 [file Supplementary_file_1.docx]

Supplementary Material

Inner speech-related brain areas mapped using a novel rhyming task

**1	Supplementary Methods**

*Covariation of reaction times (RTs) in the imaging analysis*

We run an additional analysis in which we covaried the effect of trial-wise RTs by including RT as a parametric modulator for each trial in the first level analysis. For trials without a response or with RTs below 100ms (which were considered anticipations), the participant’s mean RT in that condition was used as the value for the parametric modulator. This represented the 0.013% of all trials. The maximum number of trials with an imputed RT value for a single subject was 3. The analysis was otherwise identical to the original: GLMs were fitted to generate individual activation maps for the contrast rhyming > control and second level (group) analyses were performed within the FEAT module by means of mixed-effects GLMs. Statistical tests were carried out at the cluster level with a corrected p value of 0.05 using Gaussian random field methods. A threshold of z > 3.1, equivalent to an uncorrected p < 0.001, was used to define the initial set of clusters.

**2	Supplementary Results**

In the rhyming > control contrast, we observed a pattern of left-sided activation involving the inferior frontal cortex (*pars opercularis, pars triangularis* and *pars orbitalis*), extending into the anterior insula and Rolandic operculum. Activation was also found in the left middle and superior (posterior) temporal gyrus, supramarginal gyrus and inferior parietal cortex, and left pre- and post-central cortex. In the right hemisphere, activation was observed in the middle temporal cortex and inferior parietal/angular gyrus. We also found bilateral activation in the supplementary motor area and precuneus. The left caudate, putamen and pallidum, and the right cerebellum were also activated (Supplementary Figure S1, Supplementary Table S1).

The inverse contrast (control > rhyming) revealed significant activation in the right inferior frontal cortex, superior parietal cortex, angular and supramarginal gyrus, and superior occipital cortex. We also found significant activity in the bilateral fusiform gyrus, middle and inferior temporal areas, and the inferior and middle occipital cortex (Supplementary Figure S2, Supplementary Table S2).

**3	Supplementary Figures and Tables**

**3.1	Supplementary Figures**

**Supplementary Figure 1.** Mean brain activation map for the rhyming > control contrast when covarying trial-wise RT. Colour bar depicts z-values. Images are shown in neurological convention (right is right).

**Supplementary Figure 2.** Mean brain activation map for the control > rhyming contrast when covarying trial-wise RT. Colour bar depicts z-values. Images are shown in neurological convention (right is right).

**3.2	Supplementary Tables**

**Supplementary Table 1.** Regions of significant activation in the rhyming > control contrast when covarying for RT. MNI coordinates indicate peaks of maximum activation.

|  | **MNI coordinates** | | |  |  |  |
| --- | --- | --- | --- | --- | --- | --- |
| **Region** | ***x*** | ***y*** | ***z*** | ***Z*** | ***k*** | ***p*** |
| Left Rolandic Operculum | -58 | 10 | 4 | 5.29 | 4891 | <0.001 |
| Left inferior frontal cortex, *pars triangularis* | -38 | 26 | 2 | 4.99 |  |  |
| Left inferior frontal cortex, *pars orbitalis* | -44 | 24 | -4 | 4.82 |  |  |
| Bilateral SMA | 0 | 2 | 64 | 5.44 | 834 | <0.001 |
| Left postcentral gyrus | -56 | -8 | 50 | 4.78 | 764 | <0.001 |
| Right middle temporal cortex | 48 | -40 | -4 | 4.54 | 703 | <0.001 |
| Left putamen | -20 | 4 | -8 | 5.09 | 389 | <0.001 |
| Right lingual gyrus | 36 | -48 | 2 | 4.45 | 271 | <0.001 |
| Right cerebellum | 26 | -62 | -28 | 4.4 | 205 | 0.004 |
| Bilateral precuneus | 0 | -64 | 28 | 3.86 | 195 | 0.006 |
| Right medial prefrontal cortex | 6 | 40 | -8 | 4.01 | 187 | 0.007 |
| Right angular gyrus/inferior parietal | 64 | -58 | 32 | 4.03 | 183 | 0.008 |

SMA: Supplementary Motor Area

**Supplementary Table 2.** Regions of significant activation in the control > rhyming contrast when covarying for RT. MNI coordinates indicate peaks of maximum activation.

|  | **MNI coordinates** | | |  |  |  |
| --- | --- | --- | --- | --- | --- | --- |
| **Region** | ***x*** | ***y*** | ***z*** | ***Z*** | ***k*** | ***p*** |
| Right inferior temporal cortex | 50 | -54 | -14 | 6 | 9819 | <0.001 |
| Right angular gyrus | 28 | -66 | 48 | 5.85 |  |  |
| Right inferior occipital | 30 | -92 | -2 | 5.78 |  |  |
| Right inferior parietal | 44 | -42 | 46 | 5.59 |  |  |
| Left inferior occipital | -50 | -66 | -10 | 5.51 | 5242 | <0.001 |
| Left middle occipital | -36 | -90 | 10 | 4.92 |  |  |
| Left superior parietal | -26 | -68 | 52 | 4.85 |  |  |
| Right inferior frontal cortex, *pars opercularis* | 48 | 4 | 22 | 4.98 | 755 | <0.001 |
| Left cerebellum | -28 | -68 | -34 | 4.64 | 313 | <0.001 |
